# Ankle-brachial index and the incidence of cardiovascular events in the Mediterranean low cardiovascular risk population ARTPER cohort

**DOI:** 10.1186/1471-2261-13-119

**Published:** 2013-12-17

**Authors:** Maria Teresa Alzamora, Rosa Forés, Guillem Pera, Pere Torán, Antonio Heras, Marta Sorribes, Jose Miguel Baena-Diez, Magalí Urrea, Judit Alegre, María Viozquez, Carme Vela

**Affiliations:** 1 Primary Healthcare Centre Riu Nord-Riu Sud, Institut Català de la Salut, Santa Coloma de Gramenet, Spain; 2 Unitat de Suport a la Recerca Metropolitana Nord, Institut Universitari d’Investigació en Atenció Primària Jordi Gol (IDIAP Jordi Gol), Santa Coloma de Gramenet, Spain; 3 Primary Healthcare Centre Numància, Institut Català de la Salut, Barcelona, Spain; 4 Primary Healthcare Centre La Marina, Institut Català de la Salut, Barcelona, Spain; 5 Unitat de Suport a la Recerca de l’Àmbit de l’Atenció Primària de Barcelona Ciutat, Institut Universitari d’Investigació en Atenció Primària Jordi Gol (IDIAP Jordi Gol), Barcelona, Spain; 6 Primary Healthcare Centre Singuerlín, Institut Català de la Salut, Santa Coloma de Gramenet, Spain

**Keywords:** Peripheral arterial disease, Ankle-brachial index, Cardiovascular diseases, Incidence, Primary health care, Cohort studies

## Abstract

**Background:**

Peripheral arterial disease (PAD) of the lower limbs is a cardiovascular disease highly prevalent particularly in the asymptomatic form. Its prevalence starts to be a concern in low coronary risk countries like Spain. Few studies have analyzed the relationship between ankle-brachial index (ABI) and cardiovascular morbi-mortality in low cardiovascular risk countries like Spain where we observe significant low incidence of ischemic heart diseases together with high prevalence of cardiovascular risk factors. The objective of this study is to determine the relationship between pathological ABI and incidence of cardiovascular events (coronary disease, cerebrovascular disease, symptomatic aneurism of abdominal aorta, vascular surgery) and death in the >49 year population-based cohort in Spain (ARTPER).

**Methods:**

Baseline ABI was measured in 3,786 randomly selected patients from 28 Primary Health Centers in Barcelona, distributed as: ABI<0.9 peripheral arterial disease (PAD), ABI ≥1.4 arterial calcification (AC), ABI 0.9-1.4 healthy; and followed during 4 years.

**Results:**

3,307 subjects were included after excluding those with previous vascular events. Subjects with abnormal ABI were older with higher proportion of men, smokers and diabetics. 260 people presented cardiovascular events (incidence 2,117/100,000 person-years) and 124 died from any cause (incidence 978/100,000 person-years). PAD had two-fold greater risk of coronary disease (adjusted hazard ratio (HR) = 2.0, 95% confidence interval (CI) 1.3-3.2) and increased risk of vascular surgery (HR = 5.6, 95%CI 2.8-11.5) and mortality (HR = 1.8, 95%CI 1.4-2.5). AC increased twice risk of cerebrovascular events (HR = 1.9, 95%CI 1.0-3.5) with no relationship with ischemic heart disease.

**Conclusions:**

PAD increases coronary disease risk and AC cerebrovascular disease risk in low cardiovascular risk Mediterranean population. ABI could be a useful tool to detect patients at risk in Primary Health Care.

## Background

Prevention and early diagnosis of arteriosclerotic disease is one of the essential objectives in the field of cardiovascular disease since it is the main cause of mortality in developed countries. In Spain, cardiovascular diseases are the first cause of death, producing 32% of all deaths. Ischemic heart disease causes the greatest number of cardiovascular deaths (29%) followed by cerebrovascular disease (25%) [1].

Peripheral arterial disease (PAD) of the lower limbs is a cardiovascular disease highly prevalent particularly in the asymptomatic form. Its prevalence starts to be a concern in low coronary risk countries like Spain. Three different groups have studied it using the ankle-brachial index (ABI) with similar methodology. They have found PAD prevalence between 3.7% and 7.6% [2-5].

It is important to note that most of the PAD cases remain undiagnosed even with the presence of intermittent claudication symptoms [6]. On the other hand arterial calcification (AC), defined as ABI ≥1.4, has a prevalence of 6.2% in our country in the population > 49 years of age [7].

PAD is associated to high cardiovascular risk, in both symptomatic and asymptomatic forms. Several studies have found high incidence of cardiovascular events and mortality in patients with PAD. The MESA Study, carried out in the USA, observed a hazard ratio (HR) 1.8 to develop cardiovascular morbi-mortality in patients with PAD [8]. Ankle Brachial Index Collaboration metanalysis showed that patients with PAD had in ten years between 2 and 4 times higher risk to die or to present major cardiovascular events than patients with no PAD [9].

Although the relationship between AC and morbi-mortality has been less studied it seems to have a positive association. Ankle Brachial Index Collaboration metanalysis [9] found a moderate association with HR between 0.9 and 1.5 whereas the MESA Study observed HR 1.8 to suffer a cardiovascular event or dead in patients with AC compared with healthy patients [8].

Few studies have analyzed the relationship between ABI and cardiovascular morbi-mortality in low cardiovascular risk countries like Spain where we observe significant low incidence of ischemic heart diseases together with high prevalence of cardiovascular risk factors [10-12]. Carbayo et al. obtained HR 1.7 for cardiovascular events or death in patients with PAD with no history of previous episodes [13]. Moreover, Merino et al. showed that PAD increased the risk to suffer a major coronary episode only including men in the study regardless of previous events [14].

The objective of this study is to determine the relationship between pathological ABI and incidence of cardiovascular events (coronary disease, cerebrovascular disease, symptomatic aneurism of abdominal aorta, vascular surgery) and death in the > 49 year population-based cohort in Spain (ARTPER) [7]. Once it is confirmed an association of PAD and/or AC to increased incidence of cardiovascular events in a low cardiovascular risk country as Spain then ABI can be recommended as a simple, fast and inexpensive tool in the Primary Health Care setting to detect patients at risk.

## Methods

The ARTPER Study is an ongoing prospective observational population-based cohort study initiated in October 2006. A detailed description of the methodology of the study has been published elsewhere [15].

Briefly, at baseline ABI was measured in 3,786 randomly selected patients over the age of 49 years registered in 28 Primary Health Care centers of Barcelona region from a database including the population ascribed in the participating centers, which is even more exhaustive and updated than the census. All the subjects entered in one of the following cohorts: ABI < 0.9 peripheral arterial disease, ABI ≥ 1.4 arterial calcification, ABI between 0.9 and 1.4 healthy. An average of 4-year follow up was carried out, with phone contacts undertaken every 6 months from baseline to August 2012.

### End points

The appearance of any of the events: myocardial infarction, angina, stroke, transient ischemic attack, symptomatic aneurysm of abdominal aorta, vascular surgery (coronary, intracranial and extracranial); and vascular and overall mortality were recorded through electronic medical records, computerized clinical history, telephone interviews with the subject or with a relative, personal or telephone interview with the general practitioner in charge of the patient, the emergency departments and emergency paramedical services, and the mortality statistical records. Finally, all the events have been checked by a medical committee whose members perform routine clinical practice.

Incidence events have been grouped as follows: coronary disease (acute myocardial infarction or angina), cerebrovascular disease (stroke or transient ischemic attack), symptomatic aneurysm of the abdominal aorta (SAAA), vascular surgery, cardiovascular morbidity (any of the 4 previous ones), mortality (vascular or non-vascular cause), overall mortality and morbid-mortality (any of the mentioned events). It was only taken into account the first episode for each type of event. Any patient that had an event at the time of recruiting or a history of an event was excluded from the analysis.

### Statistical analysis

PAD and AC patient baseline profiles were separately compared to healthy patients using Chi-square test. Incidence was calculated as the number of observed events per 100,000 person-year (py), calculating Poisson 95% confidence interval (CI). Incidence of every event was associated to each of the three cohorts by means of Cox proportional hazard models, “healthy” was the reference category. Univariate associations were investigated first, and then multivariable models were carried out, adjusting by age, gender, smoking (ever = current + former), abdominal and general obesity, hypertension, hypercholesterolemia and diabetes, calculating HR and 95% CI. For each event of the study, likelihood ratio tests were used to assess the interaction of PAD (or AC) with the adjusting variables listed above. Age, abdominal and general obesity were used as continuous in Cox and interaction models. Kaplan-Meier survival function curves were done, comparing survival rates per event among the cohorts by log-rank test. A p-value less than 0.05 was considered statistically significant. Statistical analysis was performed with Stata 12.1 (StataCorp LP) software.

### Ethics

This study was approved by the local Ethics Committee (IDIAP Jordi Gol Foundation of Investigation in Primary Care and Instituto de Salud Carlos III).

Informed written consent was obtained from all the participants. Likewise, the recommendations of the World Medical Association Declaration of Helsinki were followed.

## Results

At baseline (2006–2008) 3,786 >49 year old people were enrolled representing 63% participation. Baseline prevalence of PAD and AC was 7.6% and 6.2% respectively [4]. 479 members of the ARTPER cohort previously presented some cardiovascular event and therefore were excluded from the analysis of the study. 3,307 people were enrolled to follow up, 193 (5.8%) had PAD and 198 (6.0%) AC. Demographics of the study population are shown in Table 1. 44% were men. Mean age at enrolment was 64.2 years (standard deviation (SD) 8.7, range 49–97). Patients with PAD or AC were older (6 and 2 years respectively) with higher proportion of men, smokers, hypertensive and diabetics. Patients with AC were more likely to be obese and patients with PAD more likely to present hypercholesterolemia.

**Table 1:** Baseline variables by ankle-brachial index (ABI)

	**Healthy**		**PAD**		**AC**					
	**ABI 0.9-1.4 n = 2916**		**ABI < 0.9 n = 193**		**ABI ≥ 1.4 n = 198**		**Total n = 3307**			
	**n**	**%**	**n**	**%**	**N**	**%**	**n**	**%**	p^*^	***p***^**†**^
**Gender**									0.001	<0.001
Men	1214	41.6%	105	54.4%	125	63.1%	1444	43.7%		
Women	1702	58.4%	88	45.6%	73	36.9%	1863	56.3%		
**Age at recruitment**									<0.001	0.003
49-60	1116	38.3%	38	19.7%	59	29.8%	1213	36.7%		
60-70	1084	37.2%	52	26.9%	70	35.4%	1206	36.5%		
70-97	716	24.6%	103	53.4%	69	34.8%	888	26.9%		
**General obesity**									0.112	<0.001
Underwheight/Average (BMI^**^ < 25 Kg/m^2^)	516	17.7%	43	22.3%	13	6.6%	572	17.3%		
Overweight (BMI 25–29.9 Kg/m2)	1337	45.9%	75	38.9%	86	43.4%	1498	45.4%		
Obese (BMI ≥ 30 Kg/m^2^)	1059	36.4%	75	38.9%	99	50.0%	1233	37.3%		
**Abdominal obesity** (waist circumference)^‡^									0.448	0.040
<102 cm men / <88 cm women	1144	39.5%	71	36.8%	63	32.1%	1278	38.9%		
≥102 cm men / ≥88 cm women	1749	60.5%	122	63.2%	133	67.9%	2004	61.1%		
**Smoking**									<0.001	0.007
Never smoked	1712	58.7%	82	42.5%	97	49.0%	1891	57.2%		
Ever smoked	1204	41.3%	111	57.5%	101	51.0%	1416	42.8%		
**Co-morbidity** (medical records)										
Hypertension^§^	1205	41.9%	122	64.2%	101	52.9%	1428	43.9%	<0.001	0.003
Hypercholesterolemia^_^	1283	45.0%	106	55.8%	95	48.7%	1484	45.9%	0.004	0.317
Diabetes	380	13.0%	55	28.5%	48	24.2%	483	14.6%	<0.001	<0.001

ABI: ankle-brachial index, PAD: peripheral arterial disease, AC: arterial calcification.

Individuals with prevalent events excluded.

*: *p*-value comparing ABI < 0.9 with ABI 0.9-1.4.

†: *p*-value comparing ABI ≥ 1.4 with ABI 0.9-1.4.

All comparisons made with chi-squared test.

**: BMI: body mass index.

‡ 0, 23 and 2 missing values among each ABI group.

§ 3, 41 and 7 missing values among each ABI group.

_ 3, 67 and 3 missing values among each ABI group.

**Figure 1:**
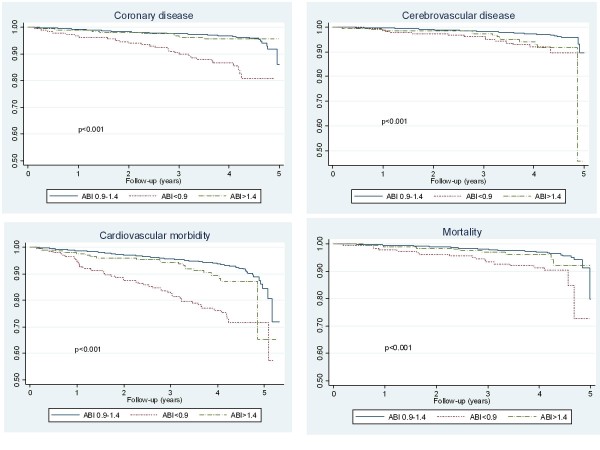
Kaplan-Meier survival curves for coronary and cerebrovascular diseases, cardiovascular morbidity and mortality among patients with different ankle-brachial index (ABI).

3,307 subjects were followed up during an average of 3.83 years (SD 0.73, range 59 days- 5.56 years, median 4.03 years) adding up 12,677 person-years. 260 participants presented cardiovascular events (incidence 2,117/ 100,000 py), among them 140 had coronary disease, 107 cerebrovascular disease, 19 SAAA and 45 vascular surgery. 124 patients died from any cause (incidence 978/ 100,000 py), 29 of them from vascular disease. A total of 347 patients suffered one cardiovascular event and/or died (Table 2). Kaplan-Meier survival curves are shown in Figure 1, being significant worse for PAD in coronary disease, cardiovascular morbidity and mortality and for AC in cerebrovascular disease.

**Table 2:** Incidence of cardiovascular events among different ankle-brachial index groups

	**Total n = 3307**				**ABI 0.9-1.4 n = 2916**				**ABI < 0.9 n = 193**								**ABI ≥ 1.4 n = 198**							
	**n**	**I**	**CI95%**		**n**	**I**	**CI95%**		**n**	**I**	**CI95%**		**HR**	**CI95%**		***p***	**n**	**I**	**CI95%**		**HR**	**CI95%**		***p***
Coronary disease	140	1124	946	1327	105	953	780	1154	27	3808	2510	5541	2.0	1.3	3.2	0.003	8	1100	475	2168	0.8	0.4	1.6	0.522
Cerebrovascular disease	107	853	699	1031	80	723	573	899	14	1895	1036	3180	1.2	0.7	2.3	0.505	13	1783	949	3049	1.9	1.0	3.5	0.051
SAAA*	19	150	90	235	14	125	69	211	4	537	146	1374	3.1	0.9	9.9	0.063	1	135	3	754	0.8	0.1	6.4	0.857
Vascular surgery	45	357	260	478	29	260	174	374	14	1941	1061	3257	5.6	2.8	11.5	<0.001	2	271	33	978	0.9	0.2	3.6	0.831
**Cardiovascular morbidity**	260	2117	1868	2391	191	1754	1514	2021	48	7104	5238	9418	2.1	1.5	2.9	<0.001	21	2935	1817	4487	1.2	0.7	1.9	0.474
**Mortality**	124	978	814	1166	95	849	687	1038	20	2657	1623	4104	1.5	0.9	2.4	0.141	9	1216	556	2308	1.0	0.5	2.0	0.947
Vascular	29	229	153	329	19	170	102	265	8	1063	459	2094	2.4	1.0	6.0	0.060	2	270	33	976	1.2	0.3	5.1	0.847
Non vascular	95	749	606	916	76	680	535	851	12	1594	824	2785	1.2	0.6	2.2	0.598	7	945	380	1948	1.0	0.5	2.2	0.947
**Morbi-mortality**	347	2826	2536	3139	263	2415	2132	2726	59	8731	6647	11263	1.8	1.4	2.5	<0.001	25	3495	3495	5159	1.0	0.7	1.6	0.838

ABI: ankle-brachial index, PAD: peripheral arterial disease, AC: arterial calcification.

*: SAAA: symptomatic aneurysm of the abdominal aorta.

Individuals with prevalent events excluded.

I = incidence per 100,000 person-year.

CI95% = 95% confidence interval of incidence (Poisson distribution) or of hazard ratio (Cox model).

HR = adjusted hazard ratio (reference ABI 0.9-1.4). HR are based on 2,799 healthy, 187 peripheral arterial disease and 189 arterial calcification subjects.

HR are adjusted by age, gender, smoking, central and general obesity, hypertension, hypercholesterolemia and diabetes.

*p* = *p*-value of HR different than 1.

PAD increased two- fold the risk of coronary disease (HR = 2.0, 95% CI 1.3-3.2) and also increased the risk of vascular surgery (HR = 5.6, 95% CI 2.8-11.5), cardiovascular morbidity (HR = 2.1, 95% CI 1.5-2.9) and morbi-mortality (HR = 1.8, 95% CI 1.4-2.5) irrespective of other cardiovascular risk factors. Although not statistically significant PAD also increased the risk to suffer SAAA and vascular mortality. AC increased twice the risk to present cerebrovascular events (HR = 1.9, 95% CI 1.0-3.5) with no relationship with ischemic heart disease (Table 2).

**Figure 2:**
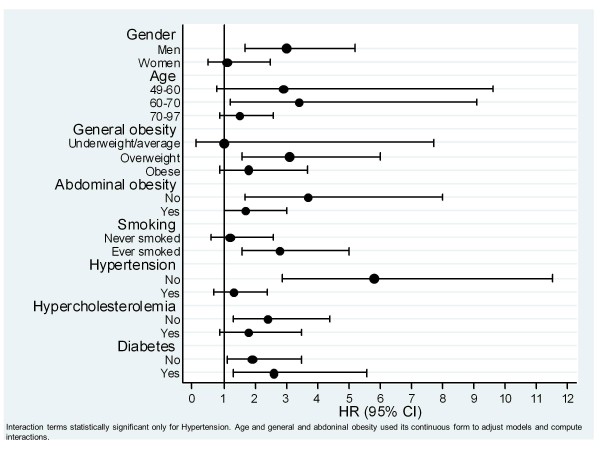
Effect of peripheral arterial disease for coronary disease, measured by hazard ratio (HR), among different potential cardiovascular risk factors.

It was observed that the effect of PAD on coronary disease was higher in men, in younger, but lower in abdominal obese, in smokers, in non-hypertensives and in diabetics. When applying interaction test this effect was only statistically significant for hypertension (Figure 2). This pattern was maintained linking PAD with cardiovascular morbidity, now with lower effects for higher body mass index. It was statistically significant the interaction of PAD with gender, hypertension and general and abdominal obese. On the other hand, PAD had a similar effect in cerebrovascular disease for each level of cardiovascular risk factors analyzed, as occurred with overall mortality (these data is not shown).

**Figure 3:**
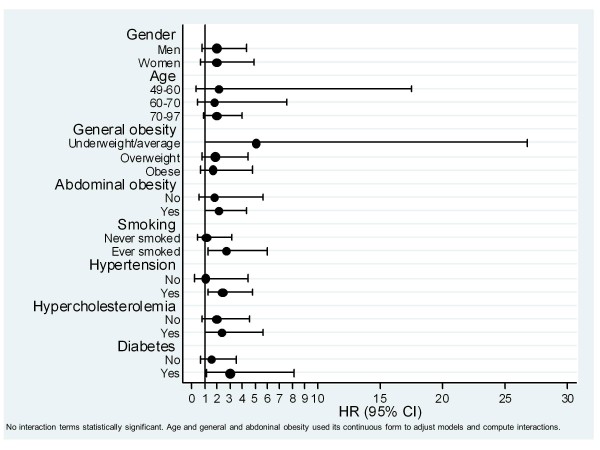
Effect of arterial calcification for cerebrovascular disease, measured by hazard ratio (HR), among different potential cardiovascular risk factors.

Higher effect was observed for AC regarding cerebrovascular disease in smokers, hypertensives, diabetics and abdominal (but not general) obese. However in no case the interaction of AC with these risk factors was statistically significant (Figure 3). AC had the same effect over coronary disease, cardiovascular morbidity and mortality across the different levels of the cardiovascular risk factors (data not shown).

High proportion of PAD patients (70%) presented asymptomatic PAD (133 patients). 56 patients (30%) presented intermittent claudication. 4 had no information. The interaction between PAD and intermittent claudication was not statistically significant to any of the studied events. However, the greatest difference of the PAD effect was found for cardiovascular morbidity. Compared to healthy, those with intermittent claudication had HR = 2.6, 95% CI 1.6-4.2, and those with asymptomatic PAD had HR = 1.7, CI 95% 1.1-2.7.

## Discussion

In the low cardiovascular risk cohort ARPER it is confirmed the importance of ABI <0.9 as an independent risk factor to have coronary disease, cardiovascular morbidity and mortality. Meanwhile ABI ≥ 1.4 is an independent risk factor to present a cerebrovascular disease.

In Spain there are no prospective studies that analyze at the same time PAD and AC as predictors of vascular events. The Albacete general population cohort [13] and the Barcelona men cohort [14] only analyzed PAD. They found similar results to our study: HR 1.9 for overall mortality and HR 3.0 for coronary disease respectively.

Our study found that patients with PAD had two-fold the risk to present a coronary event, similar results were obtained in the MESA Study (HR 1.9) [8] and in the 16 cohort ABI Collaboration metanalysis [9], (adjusted HR 2.2 for men and 2.5 for women, defining healthy as those with ABI between 1.11 and 1.40 ).

In our cohort, patients with PAD increased two- fold the risk of cardiovascular morbidity, morbi-mortality and mortality. For mortality, the higher effect was found in vascular mortality, without reaching statistical significance. ABI Collaboration metanalysis found an adjusted HR of PAD on the overall mortality 2.3 in men and 2.4 in women and greater than 3 for cardiovascular mortality [9]. The getABI Study, performed in Primary Health Care setting like the ARTPER study, obtained adjusted HR 2.0 for mortality and 1.9 for morbi-mortality in >65 year old cohort [16].

We did not observe any link between PAD and cerebrovascular disease. Same results were found in the MESA (HR 1.6) [8], ARIC (HR 1.9) [17] and Cardiovascular Health Study (adjusted HR 1.1) [18], although these results were not statistically significant. However, other studies like the German Cohort getABI [19] and the 6-study metanalysis of Heald et al. [20] observed that PAD incremented the risk to suffer cerebrovascular disease (HR of 1.8 and 1.4 respectively). Both studies recruited older patients than ARTPER. In the Heald metanalysis, the studies that found statistically significant effect of PAD on stroke were the ones that enrolled >70 year old patients.

We found association between PAD and vascular surgery and SAAA, the last with no statistical significance. These associations together to the effect observed on the coronary disease could explain the increased incidence of cardiovascular morbidity in patients with PAD. Although few studies have researched the association of PAD with vascular surgery or SAAA, their results are comparable to ours, like the getABI Study (adjusted HR 1.6 for coronary-artery revascularization in patients with PAD) [21].

With regards to AC we found an association between AC and cerebrovascular disease but not with other cardiovascular diseases or mortality. Likewise the MESA Study found greater effect of AC on stroke (HR 2.7) than on coronary disease (HR 2.2) [8]. ABI Collaboration Study obtained a link between overall mortality and AC (HR 1.3) [9], but not with coronary diseases. Whereas Sutton-Tyrrell found a relationship with coronary heart disease (HR 1.5) but not with stroke or mortality in an American cohort of >70 year old, with AC ABI cut-off of >1.3 [22].

The effect of PAD was not homogeneous among the cardiovascular risk factors. PAD seems to have greater effect on coronary disease in men, in <70 year old patients, in overweight with no abdominal obesity, smokers, diabetes, non-hypertension and dyslipidemia. It was only statistically significant the interaction of PAD with hypertension. We obtained comparable results with PAD and cardiovascular morbidity being statistically significant the interaction with gender, age and hypertension. There is scarce information in the literature about this. In our setting it has been studied the effect of PAD on cardiovascular morbidity in diabetics with similar results (HR 2.3) [23]. Nevertheless Hansen et al. did not find any difference of the effect of PAD on mortality between diabetics and non-diabetics [24].

70% of the patients with PAD were asymptomatic. This may delay the diagnosis of the disease and therefore have the risk to suffer a cardiovascular event in the future. However, similarly to the getABI Study [21], we obtained few differences of vascular events in patients with symptomatic PAD and asymptomatic PAD.

As described by other authors, the presence of PAD cannot be excluded in patients with AC because the arterial stiffness impedes a proper ABI measurement. Therefore, in our study the detection of patients with PAD and its effects could be underestimated [25,26]. In spite of that, both PAD and AC have shown an increase of cardiovascular risk and should be properly taken into account.

Patients with previous history of cardiovascular events were excluded from the study because they received secondary prevention treatment. Although these patients are at higher risk to get sick or to die [27] we preferred to concentrate our results in subjects to whom a primary prevention intervention should be done.

When patients with PAD were recruited we had to start secondary prevention treatment and therefore the effect of PAD on the cardiovascular events could be underestimated. Moreover, subjects may have changed their habits during the follow up period and prompted some misclassification attached to all longitudinal studies. These changes could be analyzed in future evaluations of the ARTPER cohort.

Our data is based on a 4 years follow-up, a period that could be too short to find significant associations between PAD and some of our cardiovascular outcomes or the interaction with other cardiovascular risk factors. However, despite our short follow-up we find relationship between PAD and coronary disease, vascular surgery, cardiovascular morbidity and morbi-mortality and between AC and cerebrovascular events, with significant interactions of PAD with gender, age and hypertension for cardiovascular morbidity.

## Conclusions

In conclusion, in the low cardiovascular risk cohort ARTPER it is confirmed the importance of PAD as an independent risk factor to present coronary disease, cardiovascular morbidity and mortality and AC as an independent risk factor to present cerebrovascular disease. The presence or absence of risk factors can modify the effect of PAD on cardiovascular diseases and this should be further studied in the future. The measurement of PAD and AC in the Primary Health Care consultation may detect high cardiovascular risk patients. Even though ABI is a simple and inexpensive instrument it will be important to define the profile of patients that are candidates to have abnormal ABI in order to have a more cost-effective screening tool.

## Abbreviations

ABI: Ankle-brachial index; PAD: Peripheral arterial disease; AC: Arterial calcification; HR: Hazard ratio; CI: Confidence interval; SAAA: Symptomatic aneurysm of the abdominal aorta; py: Person-year; SD: Standard deviation

## Competing interest

The authors declare that they have no competing interests.

## Authors’ contributions

MTA, RF, GP, PT, AH, MS, JMB, MU, JA, MV and CV participated in the design of the study. MTA, RF, AH, MS, JMB and MU contributed to the coordination study; GP participated in the statistical calculations. All the authors have read and approved the final manuscript.

## Pre-publication history

The pre-publication history for this paper can be accessed here:

http://www.biomedcentral.com/1471-2261/13/119/prepub
